# Soluble versions of outer membrane cytochromes function as exporters for heterologously produced cargo proteins

**DOI:** 10.1186/s12934-019-1270-2

**Published:** 2019-12-23

**Authors:** Helge M. Dietrich, Miriam Edel, Thea Bursac, Manfred Meier, Katrin Sturm-Richter, Johannes Gescher

**Affiliations:** 1 Department of Molecular Microbiology and Bioenergetics, Goethe University, Frankfurt, Germany; 2 Department of Applied Biology, Institute for Applied Biosciences, Karlsruhe Institute of Technology (KIT), Karlsruhe, Germany; 3 Institute for Biological Interfaces, Karlsruhe Institute of Technology (KIT), Eggenstein-Leopoldshafen, Germany

**Keywords:** *Shewanella oneidensis*, Type II secretion system, Outer membrane cytochromes, Protein export, Cellulase, Fusion proteins

## Abstract

This study reveals that it is possible to secrete truncated versions of outer membrane cytochromes into the culture supernatant and that these proteins can provide a basis for the export of heterologously produced proteins. Different soluble and truncated versions of the outer membrane cytochrome MtrF were analyzed for their suitability to be secreted. A protein version with a very short truncation of the N-terminus to remove the recognition sequence for the addition of a lipid anchor is secreted efficiently to the culture supernatant, and moreover this protein could be further truncated by a deletion of 160 amino acid and still is detectable in the supernatant. By coupling a cellulase to this soluble outer membrane cytochrome, the export efficiency was measured by means of relative cellulase activity. We conclude that outer membrane cytochromes of *S. oneidensis* can be applied as transporters for the export of target proteins into the medium using the type II secretion pathway.

## Introduction

Secretion of heterologous proteins into the culture medium is an often-used tool in biotechnology. Typically, this technique is applied in order to reduce the protein purification burden or to engineer cells to thrive on polymeric substrates via the use of excreted hydrolytic enzymes. In general, one has to distinguish between one-step and two-step export machineries. One-step export machineries transport unfolded or partially folded proteins directly from the cytosol to the medium, which requires that the protein has to fold correctly without accessory proteins like foldases or chaperons [18, 28]. Hence, the production of proteins containing cofactors is a challenge using one-step machineries. In two-step systems, the proteins are first transported across the cytoplasmic membrane via the Sec- or TAT-machinery, which allows the addition of complex co-factors and access to a machinery of proteins that aid in folding [23]. After maturation, the proteins are transported across the outer membrane by a specific secretion apparatus, the so called secretin [17, 32]. Consequently, secretion across the outer membrane is the more challenging step compared to the translocation into the periplasm. Besides these canonical one-step and two-step machineries other ways to establish transport into the culture supernatant were achieved as well. During industrial production of secreted target proteins, one commonly applied strategy is to destabilize the outer membrane by the addition of detergents which will improve the export but will also lead to a contamination with other periplasmic proteins [22]. It was also discovered that special proteins like for example YebF in *E. coli* are secreted across the outer membrane via an unknown mechanism and that a fusion to these proteins could allow the co-transport of cargo proteins [41].

Probably the best understood two-step pathway to transport folded proteins across the outer membrane is the type II secretion system (T2SS) [35]. It does not only transport mature proteins but the specific fold of the target-protein is recognized by the system and selects for transport [30]. This renders the system hard to use, as an essential recognition structure that enables interaction and transport with the T2SS has not been discovered so far and seems to differ even between closely related strains.

Besides the recognition of target proteins, secretion across the outer membrane necessitates a specific secretion apparatus [17, 32]. It consists of three major parts: an inner membrane subcomplex, several periplasmic pseudopilins and the outer membrane secretin. Interaction of all three components is necessary to enable secretion of proteins through a pore consisting of secretin protein subunits. A lipoprotein named pilotin is a facilitator protein that allows for the assembly of the secretin in the outer membrane. If pilotin is absent, the secretion system cannot operate and the secretin is degraded or mislocalized [21, 38].

Some microorganisms completely rely on the T2SS in order to thrive under certain conditions. One of these organisms is the γ-proteobacterium *Shewanella oneidensis* MR-1, which uses the T2SS for the export of outer membrane cytochromes to the cell surface [15]. These cytochromes contain multiple heme sites and are lipoproteins that form a trimeric protein complex with a β-barrel protein and a periplasmic decaheme cytochrome [13, 14]. This porin-cytochrome complex builds an electron conduit that allows the export of respiratory electrons through the outer membrane and onto insoluble electron acceptors like ferric iron or manganese oxides [5, 34]. Moreover, this extended respiratory chain is also used in biotechnological applications, as it can catalyze electron transport to and from electrodes [4, 7]. Regarding the outer membrane cytochromes it was speculated by Myers et al. that deletion of the lipid anchor leads to secretion of the proteins into the supernatant [27]. However, this was not experimentally documented yet and moreover it is not known what part of the proteins is recognized by the T2SS.

In this study we demonstrate the secretion of truncated versions of outer membrane cytochromes and asked whether protein fusions facilitate the export of heterologously expressed target proteins into the medium. For the proof of principle, we fused outer membrane cytochromes with a cellulase and measured the cellulase activity in the culture supernatant.

## Experimental procedures

### Growth conditions and media

*Escherichia coli* DH5αZI served as cloning host and was grown aerobically in LB medium at 37 °C; *S. oneidensis* strains were cultivated at 30 °C either aerobically in LB or anaerobically in M4 minimal medium (1.27 mM K_2_HPO_4_, 0.73 mM KH_2_PO_4_, 5 mM HEPES, 2 mM NaHCO_3_, 9 mM (NH_4_)_2_SO_4_, 150 mM NaCl, 3.2 mM Casamino Acids, 0.1 mM CaCl_2_, 1 mM MgSO_4_) supplemented with trace elements (5 µM CoCl_2_, 0.2 µM CuSO_4_, 5.4 µM FeCl_2_, 57 µM H_3_BO_3_, 1.3 µM MnSO_4_, 67.6 µM Na_2_EDTA, 3.9 µM Na_2_MoO_4_, 1.5 µM Na_2_SO_4_, 5 µM NiCl_2_, 1 µM ZnSO_4_). 50 mM lactate served as electron donor and 50 mM fumarate as electron acceptor. Liquid pre-cultures were cultivated oxically in LB medium at 30 °C (*S. oneidensis*) or 37 °C (*E. coli*) and 140 rpm in test tubes (5 ml medium volume). When necessary, kanamycin (50 µg ml^−1^) and arabinose (200 µM) were added to the medium.

For better cytochrome maturation and secretion, all secretion experiments were conducted under anoxic conditions. M4 medium was transferred into medium bottles (100 ml medium in 250 ml Schott flasks) or Hungate tubes (10 ml medium in 25 ml tube), respectively and the vessels were sealed with rubber stoppers. Oxygen was removed from the medium by repeatedly flushing the headspace of each bottle for 2 min with nitrogen, followed by a 2 min application of vacuum. Nitrogen gas and vacuum cycles were repeated 25 times before bottles were autoclaved. The electron donor was sterile-filtered with 0.2 µm sterile filters and added to the medium after autoclaving. After inoculation, the vessels were incubated statically at 30 °C.

### Cloning of different plasmids

The genotypes of all plasmids that were constructed in this study are listed in Table 1; all primers used for cloning are listed in Additional file 1: Table S1. In general, plasmid pBAD202 (Invitrogen, USA) served as expression vector and was linearized with *Nco*I and *Pme*I. For DNA ligations, equimolar amounts of linearized plasmid and PCR fragment with a total amount of 100 ng DNA were combined and ligation was performed using an isothermal assembly method described by Gibson et al. [19].

**Table 1:** Microbial strains used in this study

Strain name	Relevant genotype on pBAD plasmid	Reference or source
*E. coli* DH5αZI	aci q, PN25-tetR, SpR, deoR, supE44, Δ(lacZYAargFV169), Phi80 lacZDM15	[25]
Wild type		Myers and Nealson [26]
OmcA_sol_	Kan^R^, P_Ara_, *omcA*_*sol*_	This study
MtrF_sol_	Kan^R^, P_Ara_, *mtrF*_*sol*_	This study
MtrF_sol_∆22–182	Kan^R^, P_Ara_, *mtrF*_*sol*_∆22–182	This study
MtrF_sol_∆22–182_∆322–460	Kan^R^, P_Ara_, *mtrF*_*sol*_∆22–182_∆322–460	This study
MtrF_sol_Δ22–182_ Δ322–460_Linker1	Kan^R^, P_Ara_, *mtrF*_*sol*_∆22–182_∆322–460_322::Linker1::460	This study
MtrF_solΔ_22–182_ Δ322–460_Linker2	Kan^R^, P_Ara_, *mtrF*_*sol*_∆22–182_∆322–460_322::Linker2::460	This study
Cs	Kan^R^, P_Ara_, *celstrep*	This study
Cs-MtrF_sol_	Kan^R^, P_Ara_, *celstrep*_*mtrF*_*sol*_	This study
Cs-MtrF_sol_∆22–182	Kan^R^, P_Ara_, *celstrep*-*mtrF*_*sol*_∆22–182	This study
MtrF_sol_–Cs	Kan^R^, P_Ara_, *mtrF*_*sol*_-*celstrep*	This study

All strains are based on *S. oneidensis* wild type. Different versions of outer membrane cytochrome genes and fusions with the *celstrep* gene were introduced using pBAD plasmids

### Generation of soluble variants of MtrF and OmcA

In order to achieve soluble versions of MtrF and OmcA, the respective shortened DNA sequence lacking the lipid anchor was amplified from the *S. oneidensis* genome using primers 1 and 2 or primers 3 and 4, respectively. An N-terminal strep tag for immunodetection of the secreted proteins via western blot was added to the sequence of primer 2 and 4. A signal sequence (originating from the *E. coli* protein OmpA) for Sec-dependent secretion into the periplasm was amplified with primers 5 and 6 [29]. Primers of both fragments contain overlaps homologous to the vector and the corresponding fragments and were ligated into pBAD202. The resulting plasmids were named pBAD_*mtrF*_*sol*_ and pBAD_*omcA*_*sol*_, respectively.

### Construction of different MtrF_sol_ strains

For deletion of base pairs 22 to 182, primer P7 was used as a forward primer to amplify a truncated version of *mtrF* with an overlap to the *ompA* fragment, whereas P2 served as reverse primer. *OmpA* fragment was amplified as described above. Both fragments were ligated into pBAD202. The resulting plasmid variant was named pBAD*mtrF*_sol_∆22–182.

pBAD*mtrF*_sol_∆22–182 served as template for the construction of the two further variants: *mtrF*_*sol*_∆22-182_∆322–460_Linker1 and *mtrF*_*sol*_∆22–182_∆322–460_Linker2. Both were amplified via inverse PCR using primers P8 and P9 (Linker 1) or P10 and P11 (Linker 2) to add flexible linker sequences between AA321 and AA461.

### Construction of *celstrep* cellulase containing plasmids

The sequence of the cellulase gene *celstrep* was published by Amore et al. [2] and is cataloged by EMBL database number HE862416. Since the previously designed plasmids contained the *ompA*-leader sequence, the endogenous Sec-export signal of *celstrep* was redundant and omitted for further gene design. *Celstrep* was coupled to *mtrF*_*sol*_ variants to create recombinant fusion proteins. To ensure correct folding and function as well as to prevent steric interference of the two fusion protein components, their respective DNA sequences were separated by a short helical and therefore rigid spacer. This spacer descends from the Centre for Integrative Bioinformatics of the *Vrije Universiteit Amsterdam* and is cataloged by PDB number 19hc (Additional file 2: Table S2). The *celstrep* sequence was codon-optimized for *S. oneidensis* MR-1 via Java Codon Adaptation Tool (JCat) and provided by Life Technologies (Thermo Fischer, Germany) as a GeneArt™ Strings™ DNA fragment (Additional file 3: Figure S1). For C-terminal fusion into pBAD_*mtrF*_*sol*_, the synthesized sequence contained regions homologous to the plasmid. pBAD_*mtrF*_*sol*_ was linearized with *Bst*XI and integration of *celstrep* string fragment was accomplished via isothermal assembly. The spacer sequence was integrated into the sequence of primer 13. The resulting plasmid was named pBAD_*mtrF*_*sol*_-*cs*. Construction of pBAD_*mtrF*_*sol*_∆22–182-*cs* was performed analogously.

For N-terminal fusions of *celstrep* to *mtrF* variants, the *ompA* leader sequence was amplified using P5 and P6. Moreover, *celstrep* was amplified using P12 and P13, *mtrF*_*sol*_ was amplified using P14 and P2 and *mtrF*_*sol*_Δ22–182 using P15 and P2. Primers P13, P14 and P15 contain additional DNA sequences for the integration of the spacer at the N-terminus of *mtrF* variants. The fragments were ligated into pBAD via isothermal assembly and the resulting plasmids were named pBAD_*cs*-*mtrF*_*sol*_ and pBAD_*cs*-*mtrF*_*sol*_∆22–182, respectively.

### Construction of a strain expressing Celstrep cellulase without coupling to MtrF_sol_

The *celstrep* gene sequence was amplified in a PCR reaction using P16 and P17 as primers and pBAD_*cs*-*mtrF*_*sol*_ as DNA template. The resulting fragment contained an N-terminal *ompA* region and a C-terminal Strep-Tag^®^ sequence. This DNA fragment was ligated into pBAD vector as described before.

### Protein enrichment, preparation of periplasmic fractions, SDS-PAGE and Western blotting

Soluble proteins from culture supernatant were concentrated using Amicon^®^ Ultra-15 Centrifugal Filter Units (Merck-Millipore, Germany) with an exclusion volume of 3 kDa. Cells were harvested at an OD_600_ of 0.4 and the supernatant was sterile-filtered with a 0.2 µm filter. 50 ml of the supernatant were concentrated to 500 µl using centrifugal filter units at 3220×*g* and 4 °C. Periplasmic protein fractions were isolated as described [10, 31]. Protein concentrations were quantified by the method of Bradford [6], using bovine serum albumin as standard. Protein samples were separated on polyacrylamide gels [24] and the expression of the cytochromes was monitored via SDS-PAGE coupled with a heme-linked peroxidase stain [37]. Proteins containing a C-terminal *Strep*-Tag^®^ were detected on a Western blot using primary strep-tag antibodies (Qiagen, Germany) and secondary mouse antibodies labeled with alkaline phosphatase (Sigma-Aldrich, Germany). Signal detection was conducted using the AP conjugate detection kit (BioRad, Germany).

### Cellulase activity assay

Relative cellulase activity was assessed in triplicates by the method of Xiao et al. [40]. Commercially available cellulase (0.1 to 10 units ml^−1^) from *Aspergillus niger* was used to develop a standard curve.

## Results

### MtrF is a suitable exporter for protein secretion

**Fig. 1:**
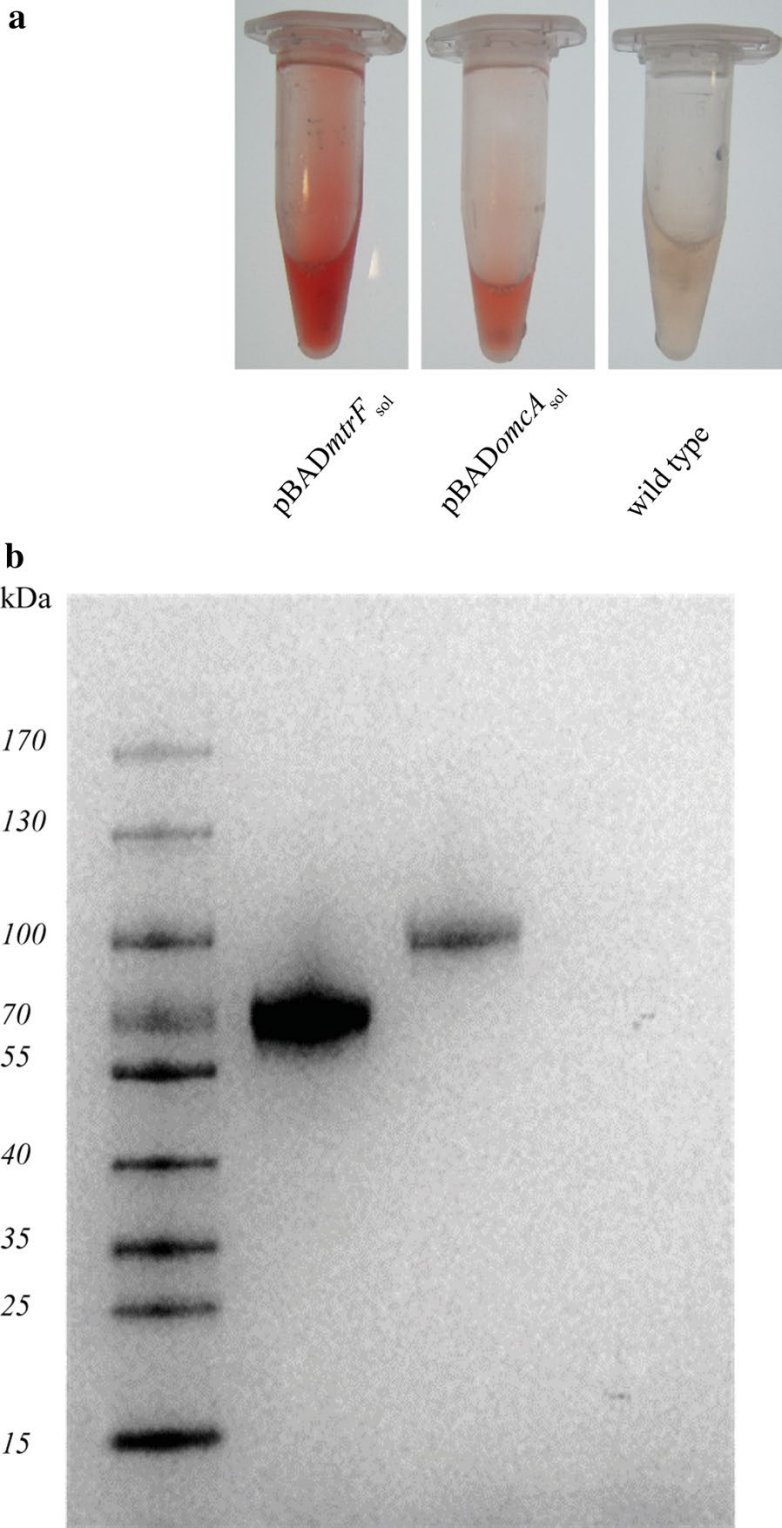
Expression of soluble OmcA and MtrF. **a** Concentrated supernatants of *S. oneidensis* expressing soluble versions of MtrF or OmcA in comparison with the wild type. Equivalent volumes were concentrated equally. The different filling levels in the tubes result from the removal of different sample volumes. The supernatant of the *mtrF*_*sol*_ expressing strain shows a more prominent red color. **b** Heme stain of an SDS gel with concentrated supernatant of *S. oneidensis* cells expressing soluble versions of MtrF or OmcA in comparison to the wild type. The supernatant of the *mtrF*_*sol*_ and *omcA*_*sol*_ expressing strains yields a strong heme signal that can be correlated to the molecular weight of the proteins

The aim of this study was to establish outer membrane cytochromes of *S. oneidensis* as transporters for export of proteins into the medium using the type II secretion pathway. Outer membrane cytochromes are attached to the outer membrane with a lipid anchor, which is connected to the protein backbone at an N-terminal cysteine residue that is part of the so-called lipobox recognition sequence. It was speculated that deletion of this lipobox sequence leads to a secretion of the truncated cytochrome into the medium [27]. Previous work revealed that of the five putative outer membrane cytochromes encoded in the genome of *S. oneidensis* only three (OmcA, MtrC and MtrF) are exposed to the cell surface [8] and are consequently potential targets for the development of a protein exporter. Still, overexpression of MtrC seems to have a more pronounced toxic effect compared to MtrF [20]. Therefore, in a first step we compared the export efficiency of soluble versions of OmcA (OmcA_sol_) and MtrF (MtrF_sol_) that lack the C-terminal lipobox sequences (amino acid deletions MtrF: Δ1–22; OmcA: Δ1–32). The corresponding genes were expressed in *S. oneidensis* wild type during anaerobic growth with lactate as electron donor and fumarate as electron acceptor and equal volumes of supernatant were analyzed for their heme signal. Anoxic conditions were used to assure identical concentration and activity of the heme maturation machinery. Both proteins hold the same number of heme centers, which is why the heme staining signals can be applied to compare the amount of exported protein. As is depicted in Fig. 1a, the concentrated supernatant of a soluble MtrF expressing culture reveals a more prominent red color and a corresponding higher signal intensity on a SDS-PAGE coupled with a heme-linked peroxidase stain compared to the identically treated samples of the wild type and the OmcA_sol_ expressing strain (Fig. 1b).

**Fig. 2:**
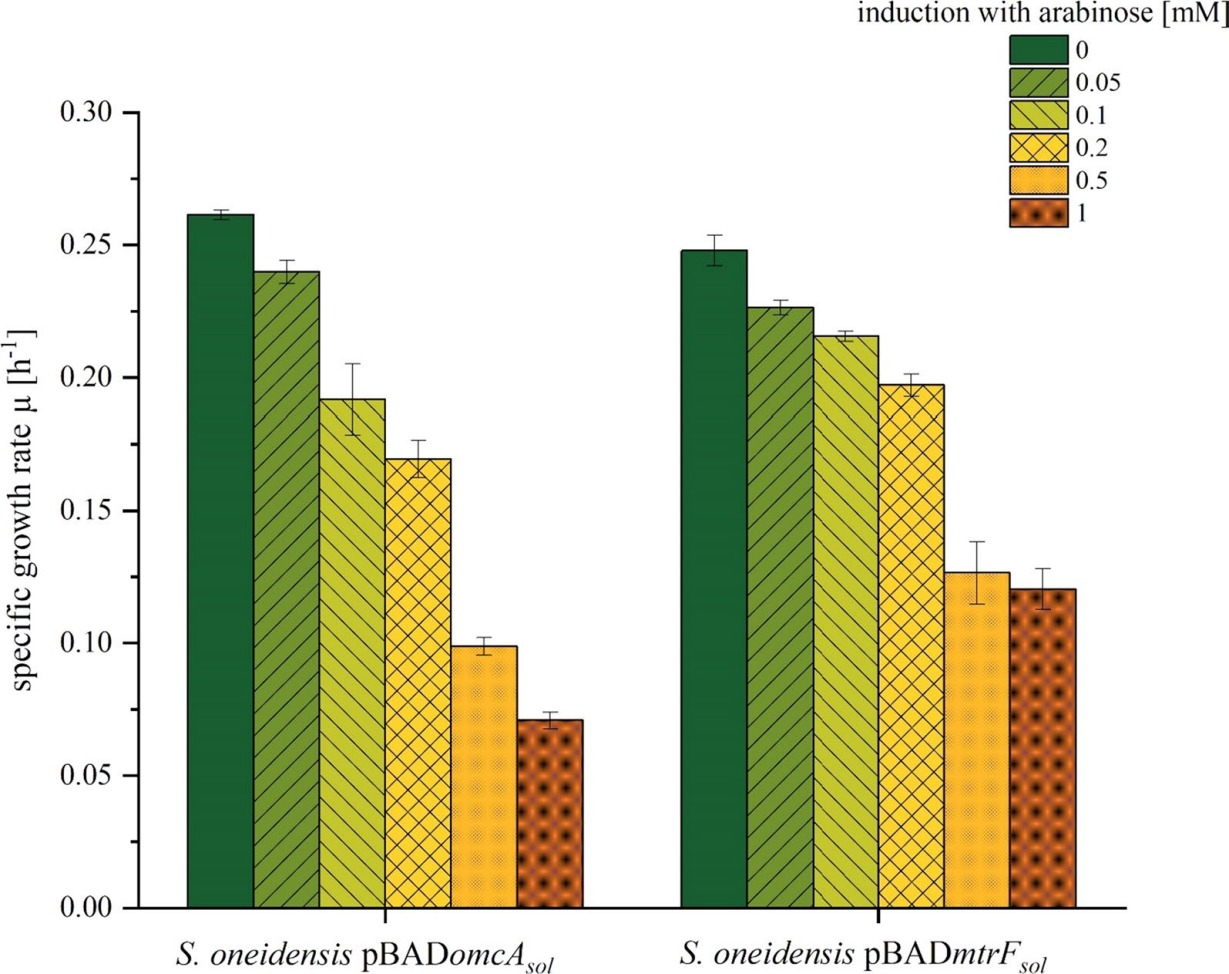
Specific growth rates of *S. oneidensis* strains with different inducer concentrations. *S. oneidensis* cultures expressing soluble versions of OmcA and MtrF, respectively, were induced with different concentrations of arabinose. Cell growth was monitored for 12 h and the specific growth rate was calculated. Error bars represent the standard deviations from means of samples taken in independent triplicates

Moreover, when we screened for potential inhibitory effects of the overexpression by comparing the growth constants under different inductor concentrations, we observed that *mtrF*_*sol*_ expression had a significantly lower impact on growth compared to *omcA*_*sol*_ (Fig. 2). Since MtrF_sol_ seems to be exported more efficiently and with less inhibitory side effects compared to OmcA_sol_, all further experiments were conducted with MtrF_sol_.

### Determination of the MtrF export signal

**Fig. 3:**
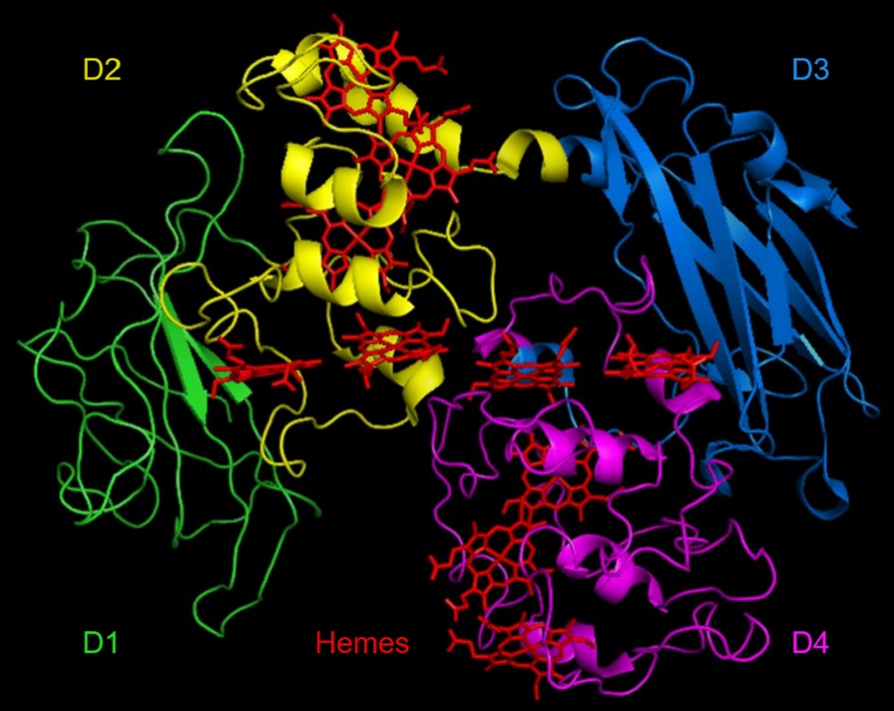
Crystal structure of MtrF. MtrF is composed of the four domains D1 (green), D2 (yellow), D3 (blue) and D4 (purple). The core domains D2 and D4 carry the ten heme groups (red) and are flanked by D1 and D3. 3D protein structures were imaged with the molecular visualization system PyMOL

The protein structure defining T2SS substrates like MtrF for translocation through the outer membrane is unknown. Our aim was to use a stepwise deletion in the MtrF_sol_ amino acid sequence to find the part of the primary structure that is relevant for recognition by the T2SS. This would increase the molecular weight ratio of cargo to exporter in the envisioned fusion proteins between MtrF_sol_ and an attached protein. The deletion strategy was developed using information from the 3D crystal structure of MtrF (PDB ID 3PMQ [12]). MtrF is composed of four domains (D1–D4). Two of these domains form a central protein core (D2 and D4) and are flanked by domains D1 and D3 (Fig. 3).

**Fig. 4:**
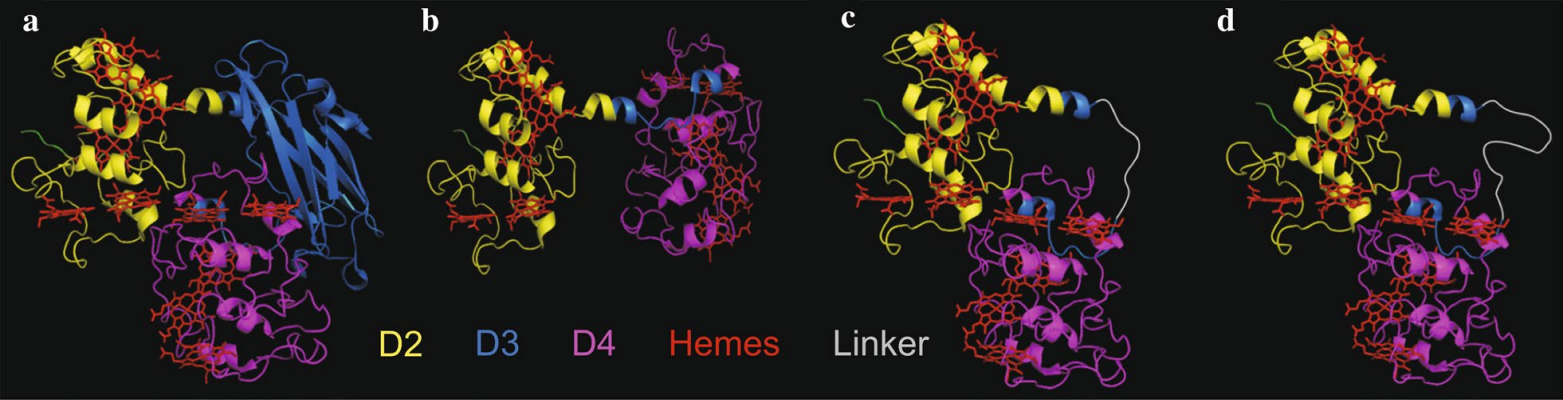
Putative structure of the developed MtrF versions. **a** MtrF_sol_∆22–182, **b** MtrF_sol_∆22–182_∆322–460, **c** MtrF_sol_Δ22–182_Δ322–460_Linker1, **d** MtrF_sol_Δ22–182_ Δ322–460_Linker2. The linker bridges the gap between domains D2 and D4. 3D protein structures were imaged with the molecular visualization system PyMOL

Partial deletions of peptide chain segments often have a far-reaching impact on protein structure and stability. Moreover, the alteration of heme coordination was shown to influence protein stability in multiheme proteins [1, 9, 16, 33]. Hence, we developed MtrF_sol_ deletion variants that are truncated as far as possible, but are not affected in heme coordination. Most of the interaction of the heme groups happen within the core domains D2 and D4. However, there are three exceptions, namely the amino acids 186, 464 and 465 that interact with hemes in the domains D1 and D3. Thus, D1 and D3 cannot be completely deleted without affecting the heme bonds. Initially, the first 182 amino acid residues (major part of D1) were deleted leading to strain MtrF_sol_∆22–182. In addition to the removal of the 182 N-terminal amino acids, deletion of amino acid 322–460 (major part of D3) was conducted, resulting in strain MtrF_sol_∆22–182_∆322–460. With these deletions it was possible to remove most of the peptide sequence without affecting the heme coordination sites of the remaining core domains. Since direct fusion of peptide fragments after removal of interjacent amino acid sequences often leads to a massive change in protein conformation, the gap between D2 and D4 was bridged with a small flexible peptide linker to maintain the original orientation and conformation of the core domains. In the original MtrF protein structure, the distance between amino acids 321 and 461 is roughly 25.5 Å. In general, flexible protein linkers often consist of glycine (G) and serine (S) residues, forming modules of (GGGGS) peptides [3, 11]. In the case of MtrF_sol_, a dimer (GGGGS)_2_ or a trimer (GGGGS)_3_ of this module named linker 1 or 2, respectively was used to bridge the gap between D2 and D4. These two peptide linkers were inserted between AA321 and AA461 in the deletion strain MtrF_sol_∆22–182_∆322–460, resulting in two strains: MtrF_sol_Δ22–182_ Δ322–460_Linker1 which has a dimer of the linker sequence inserted, and MtrF_sol_Δ22–182_ Δ322–460_Linker2 having a trimer of the linker sequence inserted (Fig. 4). Of note, the models depicted in Fig. 4 are based on the assumption that the individual structure of the remaining domains does not change after deletion of other domains, which is surely a simplification of the reality. Hence, Fig. 4 should be used as a scheme helping to understand the individual deletions and rearrangements of the primary protein structure. Further details on the DNA sequences of the linkers used in this study are described in Additional file 2: Table S2.

**Fig. 5:**
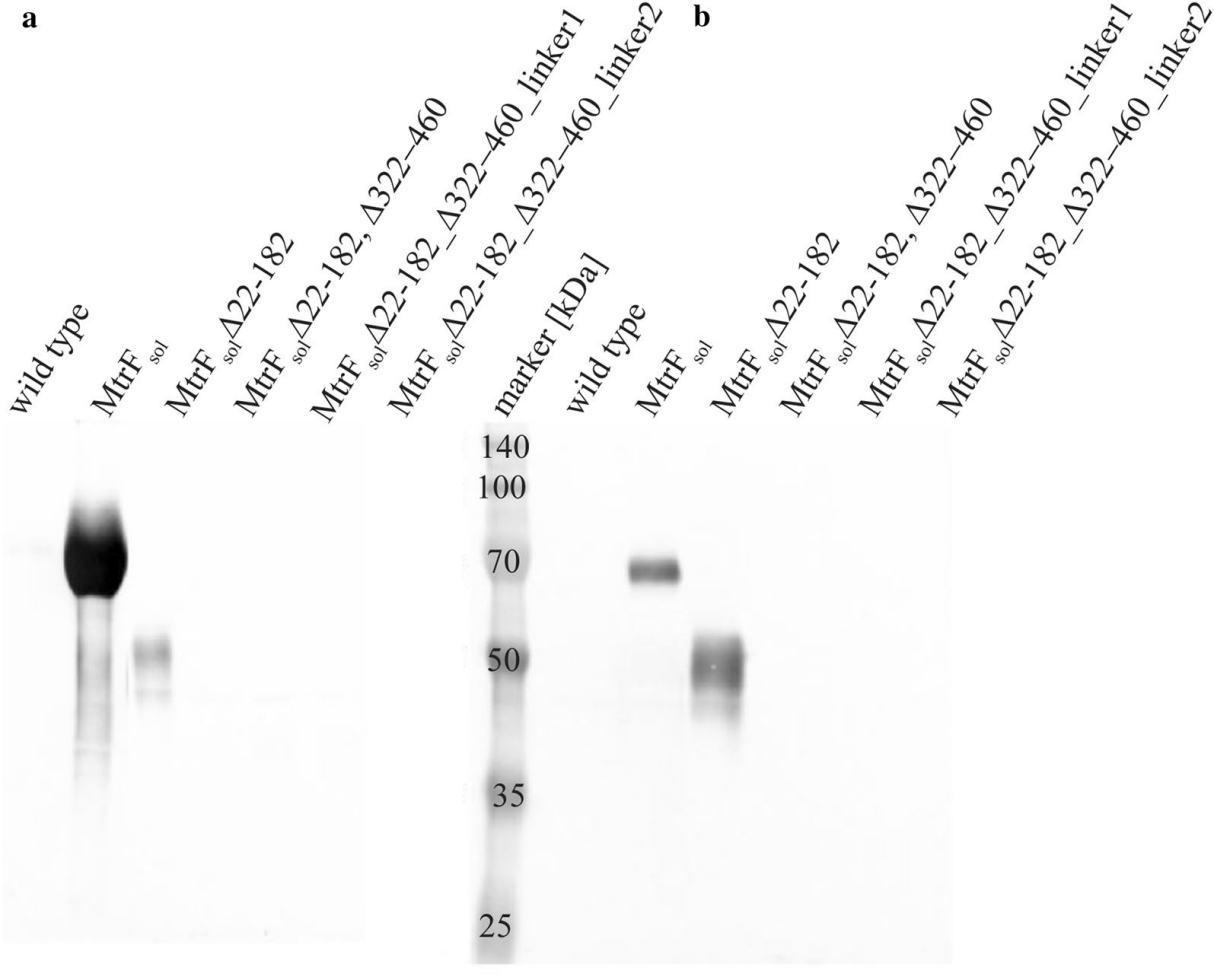
Western blot of different MtrF variants. **a** Concentrated supernatant, **b** periplasmic fraction of the MtrF versions. Only in the MtrF_sol_ and the MtrF_sol_Δ22–182 strains a signal can be detected in the supernatant and the periplasm, respectively

All constructs were expressed under identical conditions in *S. oneidensis* wild type. After the cells reached OD_600_ 0.4 the cells were harvested. The supernatant was concentrated to identical volumes and the periplasm of the cells was isolated. Western blotting analysis of the protein fractions obtained from the different strains using the N-terminal Strep-tag epitope revealed synthesis as well as secretion of MtrF_sol_ variants only for the N-terminally truncated MtrF_sol_∆22–182 (Fig. 5). Deletion of amino acids 322–460 seems to lead to instable proteins since they are neither detectable in the periplasm nor in the supernatant.

### Outer membrane cytochromes as transporter for extracellular proteins

The potential ability of MtrF_sol_ to transport other proteins into the medium was tested with the β-1,4-endoglucanase Celstrep from *Streptomyces sp.* [2]. This protein was chosen due to its potential applicability and because of the possibility to easily quantify the activity of the protein. Hence, the *celstrep* gene was coupled to the N- and C-terminus of *mtrF*_*sol*_ and *mtrF*_*sol*_∆22–182. A plasmid version containing the *celstrep* gene coupled to an N-terminal export sequence for Sec-dependent transport into the periplasm served as negative control, as this protein will not be exported from the periplasm by the T2SS. For immuno-detection, all fusion protein constructs contained a strep-tag sequence as epitope on the C-terminus.

**Fig. 6:**
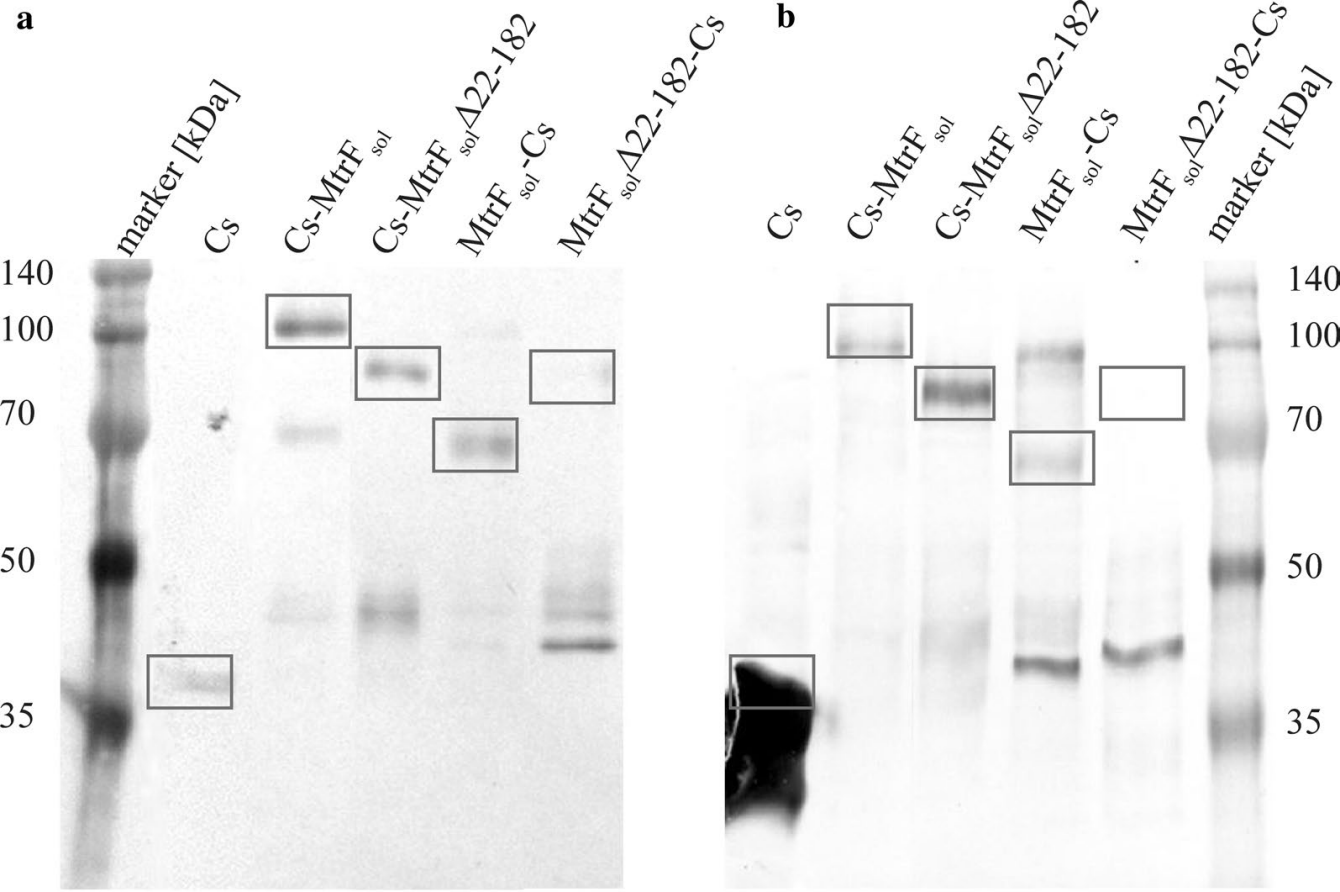
Expression of MtrF-Celstrep (MtrF-Cs) fusion proteins. Square frames indicate the size of the respective labeled proteins. Part **a** displays the concentrated supernatant and **b** the periplasmic fraction. Uncoupled cellulase can be barely found in the supernatant but there is a high amount of cellulase detected in the periplasm. The fusion proteins with the cellulase fused to the N-terminus can both be found in the supernatant and the periplasm. Full length fusion proteins with the cellulase attached to the C-terminus are barely detectable in the supernatant

As depicted in Fig. 6, the fusion proteins can be detected in the supernatant and in the periplasm in differing amounts. In agreement with the secretion experiments conducted with soluble versions of MtrF alone, we see that the full-length version seems to be exported more efficiently when fused with the cellulase, as the amount of residual fusion protein in the periplasm is lower compared to the MtrF_sol_∆22–182 versions. The Western blot also reveals the results of protein degradation by protein bands of lower molecular weight compared to the expected full-length proteins. C-terminal fusions seem to be less efficiently exported and less stable compared to the N-terminal counterparts. The control experiment with the Celstrep gene containing only a leader for the export into the periplasm reveals only a faint band in the supernatant which might be a result of cell lysis.

**Fig. 7:**
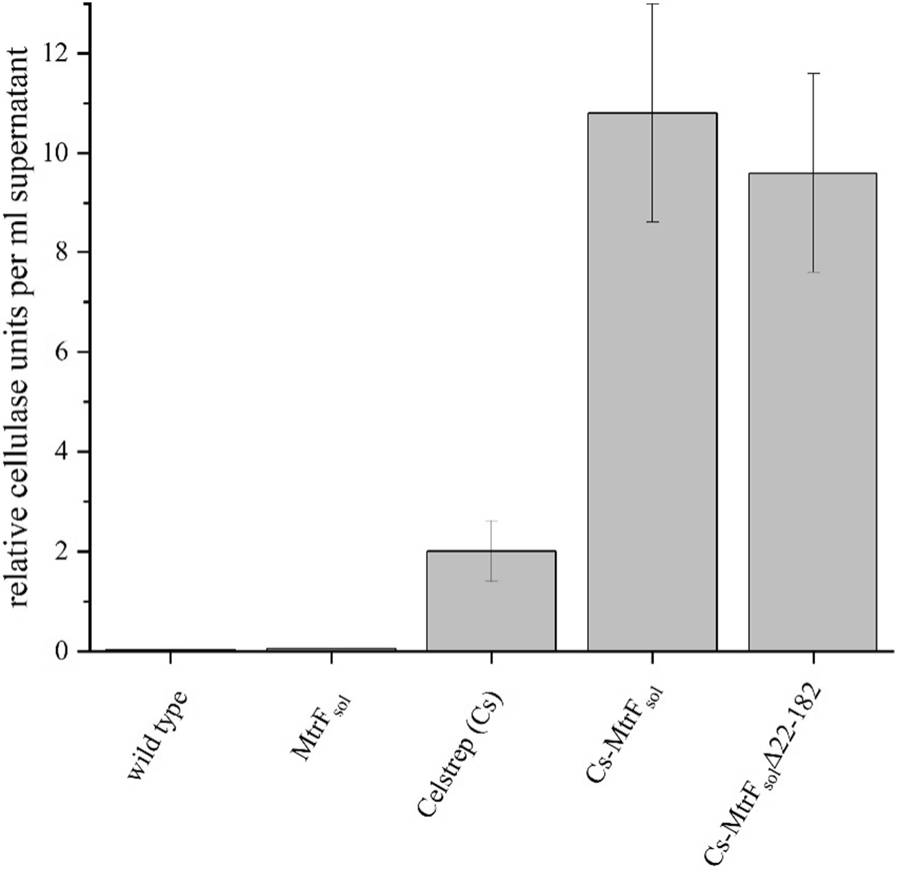
Cellulase activity of different MtrF fusion proteins. The negative controls (supernatants from wild type and MtrF_sol_ expressing cells) did not reveal any activity. The supernatant from cells expressing the cellulase only with a Sec-leader sequence for export in the periplasm (Cs) shows a rather low activity, whereas MtrF-coupled constructs are up to 3–5 times more active

An activity assay that measures the relative hydrolysis of carboxymethylcellulose (CMC) based on a method described by Xiao et al. [40] was conducted to assess the functionality of the exported proteins and to functionally compare the secretion efficiency [40]. Supernatants of *S. oneidensis* wild type cells, of cells expressing *mtrF*_*sol*_, and of wild type cells expressing N-terminal *celstrep* fusions were used for the activity analysis. Of note, only the N-terminal fusions were used as the analysis revealed a higher amount of protein in the supernatant compared to the C-terminal fusions. Supernatants were concentrated equally and their cellulase activity was measured using a calibration curve derived with a commercially available cellulase. The activity was normalized to the original volume of the supernatant. No activity was detectable in wild type and *mtrF*_*sol*_ expressing cells. The activity in the supernatant of *cs*-*mtrF*_*sol*_ expressing cells contained a cellulase activity of 10.8 (± 2.2) U/ml. The activity in the supernatant of *cs*-*mtrF*_*sol*_∆22–182 expressing cells was insignificantly lower. The supernatant of cells expressing the *celstrep* protein alone contained only 19% of the activity of the *cs*-*MtrF*_*sol*_ supernatant (Fig. 7).

## Discussion

*Shewanella oneidensis* is capable of extracellular respiration and in this process dependent on secretion of outer membrane cytochromes across the outer membrane via the T2SS. So far, it is unknown which areas of the T2SS substrates are responsible for the export information and enable the interaction with the T2SS components, and also the substrates do not show clear sequence homologies. Besides the scientific aspect to identify the secretion signal of the T2SS, there is also great interest from an applied point of view in the investigation of this export structure.

This study revealed that it is possible to secrete truncated versions of outer membrane cytochromes into the culture supernatant and that these proteins can be used as transporter for the export of heterologously produced proteins. Interestingly, no secretion was detectable after any further region of the MtrF protein besides amino acids 22–182 was deleted. All further deletions seem to be unstable as they could neither be detected in the periplasm nor in the culture supernatant. Domain D1 was identified as dispensable for a stable protein, whereas domain D3 is essential. The protein structure of this indispensable segment contains six antiparallel ß-sheet structures. Antiparallel ß-sheets are conserved among many proteins that are transported via the T2SS [36]. In MtrF, the ß-barrel structure is presumably responsible for flavin binding [12]. Our study suggests that the ß-sheets in domain D3 are of such high importance for the protein that removal of these regions leads to protein degradation.

Moreover, the results from experiments with two different versions of MtrF-CelStrep fusion proteins suggest that especially the C-terminus of the protein is relevant for protein export. A C-terminal fusion is exported to the culture supernatant barely or only to a very minor percentage, whereas an N-terminal fusion protein is secreted efficiently.

The production and the export of heterologously expressed proteins comes with a certain metabolic burden as a significant portion of cellular resources has to be allocated to this process [39]. Hence, there will be a strong selective force towards mutations since this cellular task is not connected with a physiological benefit. However, we hypothesize that the here-described tool for protein export could not only be used for a variety of cargo proteins but that it could also lead to a more stable long-term production and even to an autonomous self-advancing system. This hypothesis could be proven in an *S. oneidensis* deletion mutant that is devoid of any native outer membrane *c*-type cytochrome gene and instead capable of expressing a fusion protein consisting of a soluble outer membrane cytochrome and a cargo protein. Under conditions in which extracellular respiration is required (for example in a bioelectrochemical system), the strain has to express the soluble outer membrane cytochrome in order to thrive on the extracellular electron acceptor (the anode) and by this will benefit from cytochrome production. Furthermore, the strain will even take advantage from an evolutionary drift towards more efficient protein production and export. This self-advancing process in combination with the simultaneous production of the cargo protein could lead to a significantly enhanced protein production.

Besides the approximate localization of the secretion motif the functional integration of a rather rigid linker between the transporter and the cargo protein gives reason to assume that addition of a site-specific protease cleavage domain will be also implementable and by this it will be possible to gain the desired protein without fusion to the outer membrane cytochrome.

## Conclusions

This study demonstrates that a deletion of the lipid anchor of the outer membrane cytochromes OmcA and MtrF of the proteobacterium *S. oneidensis* leads to secretion of the truncated proteins into the culture supernatant via the T2SS. Further deletions reduced the size of secreted MtrF by 160 amino acids and revealed that for effective protein secretion the C-terminus is of particular relevance. Furthermore, a fusion of MtrF and a cellulase was secreted efficiently and gives evidence that the here-described tool for protein export can be used for a variety of cargo proteins. In this way, biotechnological applications such as production of proteins with cofactors can be optimized in terms of quality and efficiency.

## Supplementary information

**Additional file 1: Table S1.** Primer used in this study.

**Additional file 2: Table S2.** Sec leader, linker and spacer sequences used in this study.

**Additional file 3: Figure S1.** *Celstrep* DNA string. The *celstrep* gene was codon-optimized for *S. oneidensis* MR-1 and a 42 bp DNA spacer was added upstream of the gene. The sequence is flanked by homologous regions of the pBAD_*mtrFsol* plasmid, including the final base pairs of *mtrF* as well as Strep-Tag sequence (N-terminally) and downstream region of the pBAD vector (C-terminally). Additionally, the DNA string contains a *Sac*I restriction site at the beginning and a *Xba*I site at the end of the sequence.

## Data Availability

All data generated or analysed during this study are included in this published article and its supplementary information files or the datasets used and/or analysed during the current study are available from the corresponding author on reasonable request.
