# Supplementary figures and images for: Soluble versions of outer membrane cytochromes function as exporters for heterologously produced cargo proteins
Source: Microb Cell Fact. 2019 Dec 23;18:216. doi: 10.1186/s12934-019-1270-2 (PMC6929479; doi:10.1186/s12934-019-1270-2)

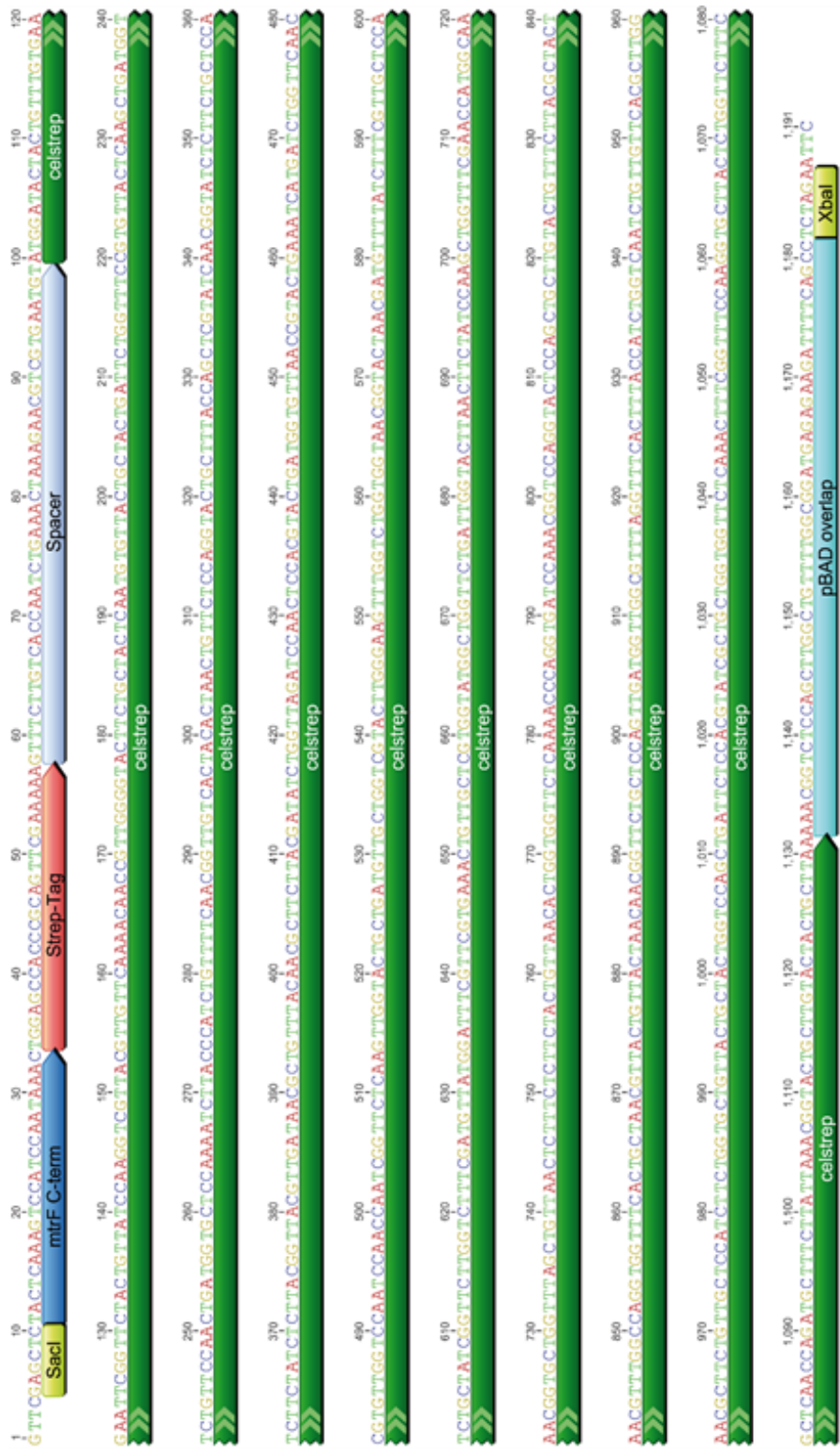

Supplement: Supplementary file 3 — Additional file 3: Figure S1. Celstrep DNA string. The celstrep gene was codon-optimized for S. oneidensis MR-1 and a 42 bp DNA spacer was added upstream of the gene. The sequence is flanked by homologous regions of the pBAD_mtrFsol plasmid, including the final base pairs of mtrF as well as Strep-Tag sequence (N-terminally) and downstream region of the pBAD vector (C-terminally). Additionally, the DNA string contains a SacI restriction site at the beginning and a XbaI site at the end of the sequence. [file 12934_2019_1270_MOESM3_ESM.pdf]
